# Potent and Specific Antitumor Effect for Colorectal Cancer by CEA and Rb Double Regulated Oncolytic Adenovirus Harboring *ST13* Gene

**DOI:** 10.1371/journal.pone.0047566

**Published:** 2012-10-15

**Authors:** Xiumei Zhou, Guoliang Xie, Shibing Wang, Yigang Wang, Kangjian Zhang, Shu Zheng, Liang Chu, Lianli Xiao, Yuemei Yu, Yue Zhang, Xinyuan Liu

**Affiliations:** 1 Xinyuan Institute of Medicine and Biotechnology, College of Life Science, Zhejiang Sci-Tech University, Hangzhou, China; 2 State Key Laboratory of Cell Biology, Institute of Biochemistry and Cell Biology, Shanghai Institutes for Biological Sciences, Chinese Academy of Sciences, Shanghai, China; 3 Department of Laboratory Medicine, The First Affiliated Hospital, College of Medicine, Zhejiang University, Hangzhou, China; 4 Cancer Institute, The Second Affiliated Hospital, College of Medicine, Zhejiang University, Hangzhou, China; Vanderbilt University, United States of America

## Abstract

Cancer Targeting Gene-Viro-Therapy (CTGVT) is constructed by inserting an antitumor gene into an oncolytic virus (OV). It is actually an OV-gene therapy, which has much better antitumor effect than either gene therapy alone or virotherapy alone in our previously published papers. This study is a modification of CTGVT by inserting a colorectal cancer (CRC) specific suppressor gene, ST13, into a CRC specific oncolytic virus, the Ad·CEA·E1A(Δ24), to construct the Ad·(ST13)·CEA·E1A(Δ24) for increasing the targeting tropism to colorectal cancer and it was briefly named as CTGVT-CRC. Although many studies on CEA promoter and ST13 gene were reported but no construct has been performed to combine both of them as a new strategy for colorectal cancer (CRC) specific therapy. In addition to the CRC specificity, the antitumor effect of Ad·(ST13)·CEA·E1A(Δ24) was also excellent and got nearly complete inhibition (not eradication) of CRC xenograft since ST13 was an effective antitumor gene with less toxicity, and a Chinese patent (No. 201110319434.4) was available for this study. Ad·(ST13)·CEA·E1A(Δ24) caused cell apoptosis through P38 MAPK (i.e. P38) which upregulated CHOP and ATF2 expression. The mitochondrial medicated apoptosis pathway was activated by the increase of caspase 9 and caspase 3 expression.

## Introduction

Cancer is a major global public health concern. A total of 1,529,560 new cancer cases and 569,490 deaths from cancer occurred in the United States alone in 2010 [1]. Colorectal cancer is the second highest cause of death in the USA and is the fourth most common cancer in men and the third most common cancer in women worldwide [2]. Thus, it is essential for scientists and medical doctors to develop new strategies for colon cancer treatment. One strategy that was initiated by us in 1999 through 2011, termed Cancer Targeting Gene-Viro-Therapy (CTGVT), involves the insertion of an antitumor gene into an oncolytic virus (OV) [3], [4]. It is actually an OV-gene therapy. The CTGVT (OV-gene) has potent antitumor effect, which is the result of the inserted genes to be replicated several-hundred fold along with the replication of the oncolytic virus in cancer cells [5]. Usually, the order of antitumor effect is better by CTGVT (OV-gene) than the effect by OV and Ad-gene. We have devoted ourselves to study the CTGVT (OV-gene) strategy for over 10 years and published about 70 related papers, which always showed much higher antitumor activity than that of Ad-gene [6], [7], [8]. The CTGVT (OV-gene) is timely becoming a hot topic since Amgen paid 1 billion USD to purchase the OncoHSV-GM-CSF (OV from Herpes Simplex Virus) from BioVex [9] and the OncoPox-GM-CSF has been published in Nature, 2011 [10].

Colorectal tumorigenesis is a complicated process that is driven by multiple genes and involves numerous steps. Previous research has shown that *ras* gene mutations; deletions in chromosomes 5q, 17q and 18q; *neu, c-myc,* or *c-myb* amplifications; and rearrangements of the *trk* oncogene were involved in colorectal tumors [11]. However, these molecular changes could not fully explain the entire process of colorectal tumorigenesis. In 1993, Zheng *et al*. identified a colorectal cancer-related gene that was downregulated in colorectal cancer, named suppression of tumorigenicity 13 (ST13) (GenBank accession No. HSU17714), which was cloned by subtractive hybridization screening between the cDNA of normal mucosal tissues and the mRNA of colorectal carcinoma tissues [12], [13], [14], [15]. Thus, ST13 was a candidate tumor-suppressor gene involved in colorectal carcinoma [16], [17]. Increased ST13 protein expression could suppress proliferation and induce the apoptosis of colorectal cell lines. Our previous research verified that the use of ZD55-ST13 (a oncolytic adenovirus deleting E1B 55KDa) led to a 100-fold inhibitory effect for tumor cell growth compared to Ad-ST13 *in vitro,* and ZD55-ST13 also exerted a potent antitumor effect in an SW620 xenograft animal model of colorectal carcinoma [18]. The improved antitumor efficacy of another oncolytic adenovirus construction SG500-ST13 over SG500 was apparent from experiments using the HCT116 and SW620 cell lines *in vitro* as well as the application of the HCT116 xenograft model *in vivo* [19]. In all the above ST13 research, there is no work using the cell type-specific CEA promoter to drive the E1A(Δ24) expression to control selective replication of virus for further CRC specific therapy.

Carcinoembryonic antigen (CEA) is a widely used tumor marker, especially in the surveillance of colorectal cancer patients [20]. Recent experiments indicated that CEA may function as a cell adhesion molecule that could play an important role during embryogenesis and possibly during tumor development [21]. Schrewe H *et al*., found that a CEA gene promoter construct demonstrated anticancer activity that was nine times greater against the CEA-producing adenocarcinoma cell line SW403 than the non-CEA-producing HeLa cell line [20]. The CEA promoter coupled to the herpes simplex virus thymidine kinase (HSV-tk), appeared to selectively upregulate the expression of HSK-tk in the CEA-expressing pancreatic carcinoma cell line BXPC3, which led to antitumor effects [22]. AdCEAp/Rep, in which E1A expression was driven by the CEA promoter, effectively inhibited multiple liver metastases of the CEA-positive colon cancer M7609 in a xenograft model [23].

In this study, ST13 gene was inserted into an oncolytic viral vector Ad·CEA·E1A(Δ24) that applied CEA promoter to control E1A expression with a 24-bp deletion in the E1A region responsible for binding retinoblastoma (Rb) protein and its replication and this construct was referred to as Ad·(ST13)·CEA·E1A(Δ24) (the ST13 in the parenthesis represents an expression cassette). This is a modification of CTGVT with CRC specific Cancer Targeting Gene-Viro-Therapy (briefly CTGVT-CRC), which constitutes a novel strategy that has not been previously reported. Our data indicated that the antitumor of Ad·(ST13)·CEA·E1A(Δ24) was higher than that of Ad·(EGFP) CEA·E1A(Δ24), further higher than that of ONYX-015 *in vitro* and *in vivo*. Ad·(ST13)·CEA·E1A(Δ24) treatment significantly inhibited but not completely eradicated the growth of xenograft SW620 colorectal carcinomas in nude mice, and the survival time was dramatically improved without one nude mice death in the Ad·(ST13)·CEA·E1A(Δ24) treated group. These results provide a novel insight for clinical colorectal cancer-specific therapy and a patent has been applied [201110319434.4].

## Materials and Methods

### Plasmids, Virus and Reagents

The pMD18-T vector was purchased from TaKaRa, Ltd., and the pBHGE3 plasmid was purchased from Microbix Biosystem Inc. (Toronto). The pAd·E1A(Δ24), pMD18-T Simple-HCMV-MCS-polyA(SV40) and pXC2-CEA plasmids were previously constructed by our group. The pCA13-ST13, ONYX-015 and Ad-WT viruses were maintained in our laboratory.

**Figure 1:**
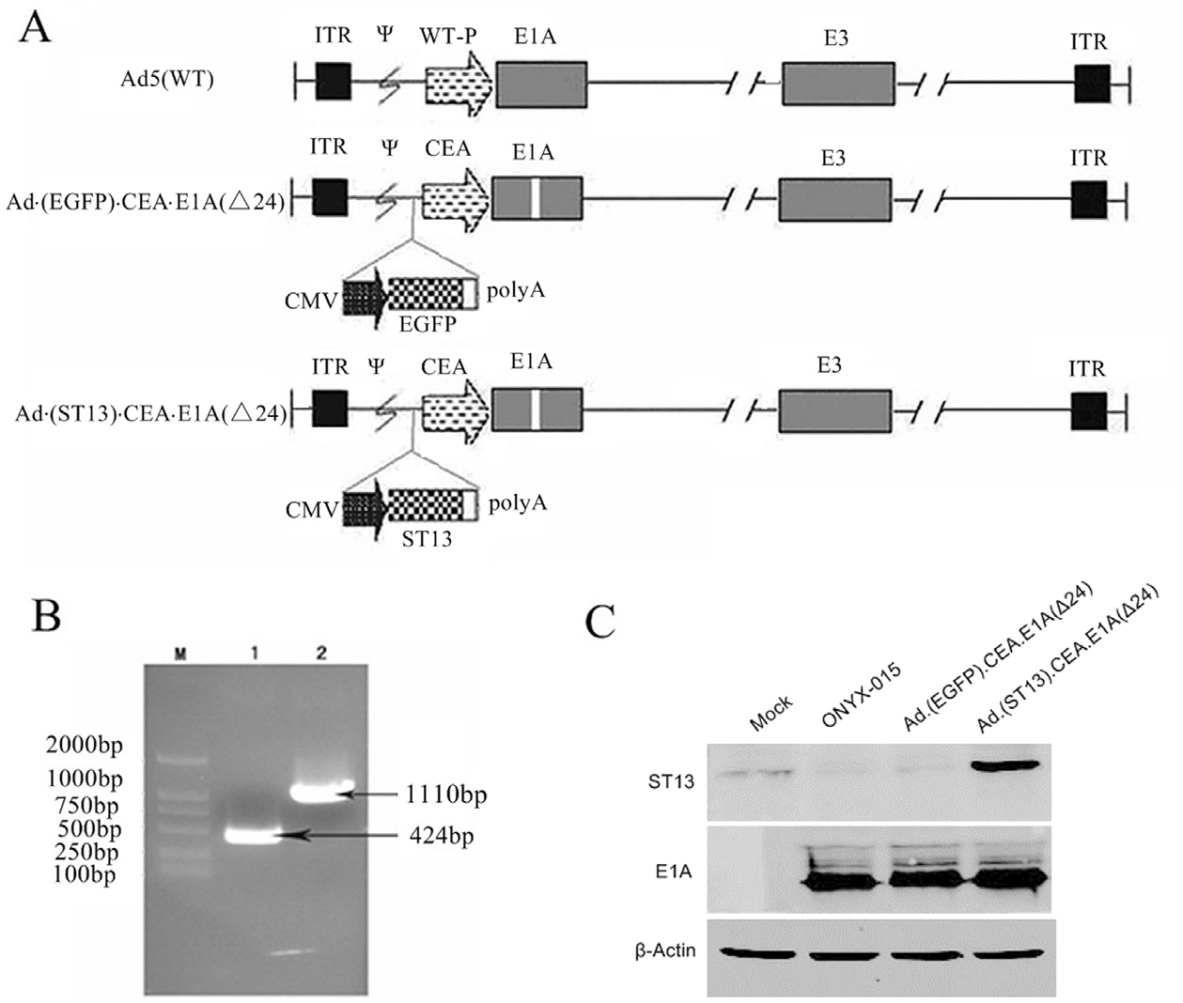
Construction and characterization of Ad·(ST13)·CEA·E1A(Δ24).(ST13) represents the expression cassette. **A.** Schematic construction of Ad·(ST13)·CEA·E1A(Δ24) and Ad·(EGFP)·CEA·E1A(Δ24). The native *E1A* promoter was replaced by the CEA promoter to control the expression of the *E1A* gene with a 24-bp deletion. An ST13 or EGFP expression cassette under the control of the hCMV promoter was inserted between the ψ packing signal sequence and the E1A gene. **B.** Identification of the CEA promoter and ST13 gene in pAd·(ST13)·CEA·E1A(Δ24) by PCR. Lane M: DL2000 Marker; Lane 1: CEA promoter; Lane 2: ST13 gene. **C.** Detection of E1A(Δ24) and ST13 expression levels when SW620 cells were infected with Ad·(ST13)·CEA·E1A(Δ24), Ad·(EGFP)·CEA·E1A(Δ24) or the typical oncolytic virus ONYX-015 at an MOI of 5 for 48 hr. Western blot analysis was conducted to detect E1A(Δ24) and ST13 protein levels.

Antibodies against caspase-3, caspase-9, Fas, Bcl-XL, CHOP, E1A, p38, Phospho-p38 MAP Kinase, ATF-2 and Phospho-ATF-2 were purchased from Cell Signaling Technology, Inc. The PARP-1/2 and β-actin antibodies were obtained from Santa Cruz Biotechnology (Santa Cruz, CA, U.S.A), anti-ST13 and anti-Hexon antibodies were purchased from Epitomic, and the IRDye® 680 donkey anti-mouse IgG and IRDye® 680 donkey anti-rabbit IgG were purchased from LI-COR.

**Figure 2:**
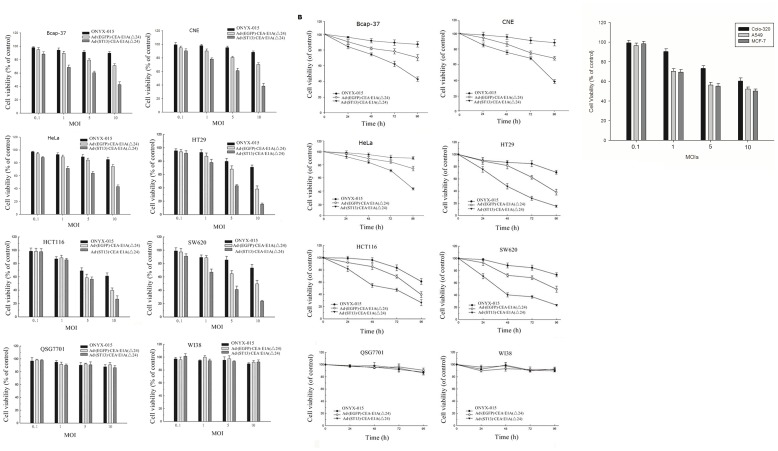
Colorectal cancer specific antitumor effect of Ad·(ST13)·CEA·E1A(Δ24) *in vitro* analyzed by the MTT assay. A. The viability of tumor cells infected with different MOIs of the various oncolytic adenoviruses. Three CRC tumor cell lines (SW620, HCT116 and HT29), and three CEA-negative cell lines (Bcap37 breast cancer, CNE Nasopharynageal carcinoma and HeLa cervical carcinoma) and two normal cells (QSG7701 and WI38) were infected with either Ad·(ST13)·CEA·E1A(Δ24), Ad·(EGFP)·CEA·E1A(Δ24), or the typical oncolytic virus ONYX-015 at a range of MOIs (0.1, 1, 5 or 10 MOI), 4 days, cell viability was determined using an MTT assay. Uninfected cells were considered to be 100% viable. Bars represent the means ± SD (n = 6). **B.** The influence of viral infection on cell viability at different times. Three CEA positive cell lines (SW620, HCT116, and HT29) and three CEA-negative cell lines (Bcap37, CNE and HeLa) and two normal cells (QSG7701 and WI38) were infected with either ONYX-015, Ad·(EGFP)·CEA·E1A(Δ24), or Ad·(ST13)·CEA·E1A(Δ24) at an MOI of 10. After 24, 48, 72, and 96 hours, the cell viability was measured using the MTT assay. The data are presented as the mean ± SD of triplicate experiments. **C.** The viability of tumor cells infected with different MOIs of Ad·(ST13)·CEA·E1A(Δ24). CEA-negative colon cancer cell line (Colo-320) and CEA-positive non-colon cancer cell line (A549, MCF-7) were infected with Ad·(ST13)·CEA·E1A(Δ24) at a range of MOIs (0.1, 1, 5 or 10 MOI), 3 days, cell viability was determined using an MTT assay. Bars represent the means ± SD (n = 6).

**Construction of different recombinant adenoviruses**The CEA promoter from pXC2-CEA was subcloned into pAd·E1A(Δ24) to form pAd·CEA·E1A(Δ24) carrying the adenovirus serotype 5 E1A gene with a 24-bp deletion from 923 bp to 946 bp. The entire ST13 expression cassette was further inserted into pAd·CEA·E1A(Δ24) to form pAd·(ST13)·CEA·E1A(Δ24). The construction method for the generation of pAd·(EGFP) CEA·E1A(Δ24) was similar to that for pAd·(ST13) CEA·E1A(Δ24)_._ The sequence of each of the plasmid constructs was confirmed using restriction enzyme digestion, PCR and DNA sequencing. The oncolytic viruses Ad·(EGFP)·CEA·E1A(Δ24) and Ad·(ST13)·CEA·E1A(Δ24) were generated by homologous recombination between pAd·(EGFP)·CEA·E1A(Δ24) or pAd·(ST13)·CEA·E1A(Δ24) with the adenovirus packaging plasmid pBHGE3 (Microbix Biosystem) in HEK293 cells using the effectene transfection reagent (Qiagen, Hilden, Germany) according to the manufacturer’s instructions. Each recombinant adenovirus was selected after three rounds of plaque purification in HEK293 cells and was separately identified by PCR. The final purification of the virus was performed by cesium chloride density gradient centrifugation.

**Figure 3:**
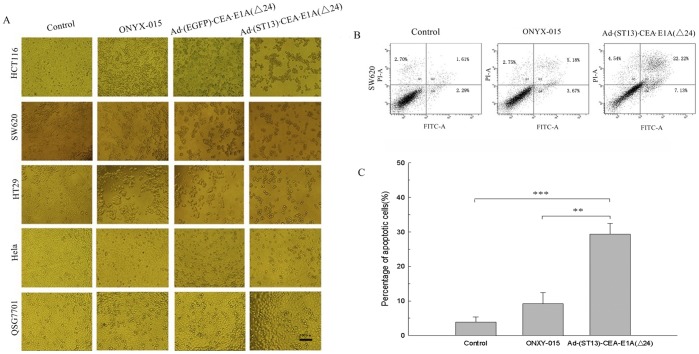
Morphological changes and apoptosis detected by flow cytometry. A. Morphological observations of tumor cells and normal cells infected with the various oncolytic adenoviruses as detected by microscopy. Cells were infected at an MOI of 10, and the morphological changes in the cells were observed by microscopy after 72 hours of infection. **B.** Detection of apoptosis in SW620 cells by FACS. SW620 cells were infected with either ONYX-015, Ad·(EGFP)·CEA·E1A(Δ24) or Ad·(ST13)·CEA·E1A(Δ24) at an MOI of 10. At 48 hours, the cells were harvested and stained with annexin V-FITC (for early-stage apoptosis) or PI (for late-stage apoptosis) and were examined by flow cytometry. **C.** The percentage of apoptotic cells was calculated using the Cell Quest software. The data are presented as the mean ± SD (error bars) of triplicate experiments. (**p<0.01; ***p<0.001).

### Cell Lines and Cell Culture

HEK293 (human embryonic kidney cell line) cells, A549 (human lung adenocarcinoma line), Colo-320(human colon cancer cell line), and MCF-7(human breast cancer cell line) were obtained from the American Type Culture Collection (ATCC, Rockville, MD, USA). HeLa (human cervical carcinoma cell line), SW620, HT29 and HCT116 (human colorectal cancer cell lines), WI38 (human normal fetal lung fibroblast cell line), and QSG-7701 (human normal liver cell line) cells were purchased from the Cell Bank at the Type Culture Collection of the Chinese Academy of Sciences (Shanghai, China). SW620 cells were cultured in Dulbecco’s Modified Eagle’s Medium (DMEM, GIBCO BRL, Grand Island, NY) that was supplemented with 5% fetal bovine serum (FBS), 4 mM glutamine, penicillin (100 U/mL), and streptomycin (100 µg/mL). All of the other cell lines were cultured in DMEM supplemented with 10% FBS. All of the cell cultures were maintained at 37°C with 5% CO_2_ in a humidified incubator. Cells in a logarithmic growth phase were used for the experiments.

**Figure 4:**
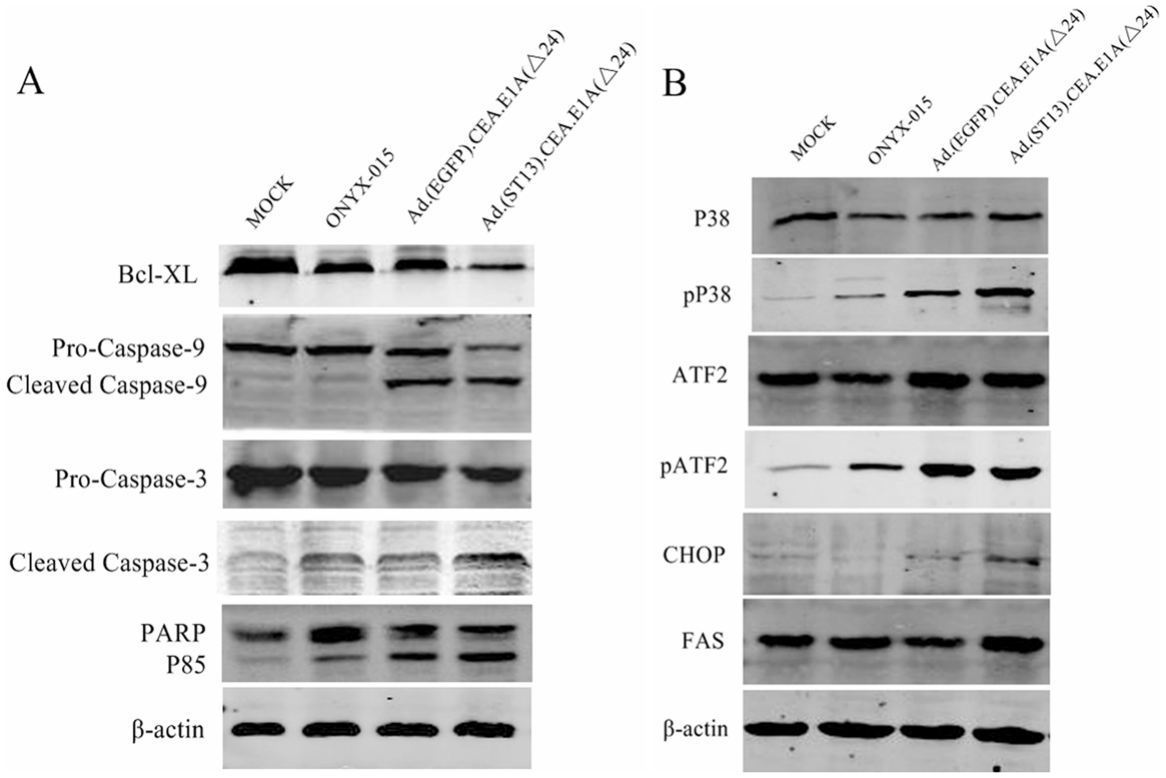
Detection of apoptosis in SW620 **cells by western blot.** SW620 cells were infected with either ONYX-015, Ad·(EGFP)·CEA·E1A(Δ24) or Ad·(ST13)·CEA·E1A(Δ24) at an MOI of 5, for 48 h, the apoptosis-related proteins were analyzed by western blot.

### Western Blot Analysis

To further explore the molecular mechanisms responsible for the cell death induced by the recombinant adenovirus, Western blot analyses were performed. SW620 and HCT116 cells were seeded into 6-well plates at a density of 5×10^5^ per well and cultured overnight, and they were then treated with or without the virus for 48 h. Both adherent and floating cells were harvested in lysis buffer [62.5 mM Tris-HCl (pH 6.8), 2% sodium dodecyl sulfate (SDS), 10 mM glycerol, and 1.55% dithiothreitol]. Protein concentrations were determined using a Bicinchoninic Acid (BCA) protein assay kit (Thermo Scientific, Rockford, USA). Equal amounts of proteins were separated by 12% SDS-PAGE and were then transferred to nitrocellulose (NC) membranes. The membranes were blocked with 5% skim milk and then incubated with primary antibodies and the appropriate secondary fluorescent antibodies. Immunodetection was visualized using an Odyssey infrared imaging system (LI-COR Biosciences Inc, America).

### Cell Viability Assay

Cells were dispensed into 96-well plates and treated with either ONYX-015, Ad·(EGFP)·CEA·E1A(Δ24), or Ad (ST13)·CEA·E1A(Δ24) at the indicated MOIs and time points. An MTT assay was conducted to determine cell viability following treatment with the various adenoviruses. Four hours before the end of the incubation, 20 µL of MTT solution (5.0 mg/mL) was added to each well. The resulting crystals were dissolved with 150 µL DMSO/well by shaking for 10 min. The optical density (O.D.) was measured at 570 nm using a DNA microplate reader (GENios model; Tecan, Mannedorf, Switzerland). The cell survival percentage was calculated using the following formula: cell survival % = (absorbance value of treated cells/absorbance value of untreated control cells)×100%. Six replicate samples were evaluated for each concentration.

**Figure 5:**
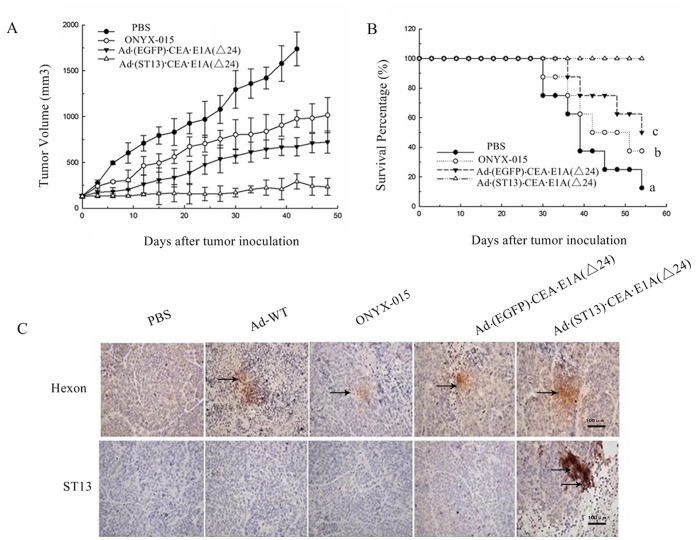
The antitumor efficacy of Ad·(ST13)·CEA·E1A(Δ24) in nude mice bearing a colorectal cancer SW620 xenograft. Tumors were established by injecting SW620 cells subcutaneously into the right flank of nude mice. When tumors reached 100–130 mm^3^, the mice were randomly divided into three groups (n = 8) and were treated daily with consecutive intratumoral injections four times of ONYX-015, Ad·(EGFP)·CEA·E1A(Δ24) or Ad·(ST13)·CEA·E1A(Δ24) at 5×10^8^ PFU/day and PBS. **A.** The tumor size was measured with calipers, and the tumor volume was calculated using the following formula: tumor volume (mm^3^) = 0.5×length×width^2^. **B.** The survival curve for the animals during the observation period. The data are presented as the mean ± SD (error bars). A log-rank test has been used to analyze survival rates in the different groups. Statistical significance: a, p<0.001, compared with PBS; b, p<0.01, compared with ONYX015; c, p<0.05, compared with Ad*(EGFP)*CEA*E1A (Δ24). **C.** Hexon and ST13 expression *in vivo.* Tumor sections derived from PBS- or different adenovirus drugs treated 4 days were analyzed for Hexon and ST13 expression by immunohistochemistry. Original magnification 400x.

### Flow Cytometry Analysis

Human colorectal cancer SW620 cells were seeded in 6-well plates at a density of 5×10^5^ per well and were cultured at 37°C with 5% CO_2_ in a humidified incubator. Following overnight culture, the cells were treated with either ONYX-015 or Ad·(ST13)·CEA·E1A(Δ24) at an MOI of 5. The cells were trypsinized and harvested 48 h after treatment. The cells were then stained with annexin V-fluorescein isothiocyanate (FITC) and propidium iodide (PI) in a binding buffer, as described in the annexin V-FITC apoptosis detection kit protocol (BioVision, Palo Alto, CA). After staining, the cells were analyzed for apoptosis using fluorescence-activated cell sorting (FACS; Becton Dickinson).

### Ethics Statement and Animal Experiment

Male BALB/c nude mice (4-week-old) were maintained and used in a light and temperature controlled room in an AAALAC-accredited facility, and given water and lab chow *ad libitum.* All experimental procedures were approved by the Institutional Animal Care and Use Committee of Shanghai Institute of Biochemistry and Cell Biology under protocol IBCB-SPF0029. Xenografted mice were used as a model system to study the cytotoxic effects of SW620 cells (Chinese Academy of Sciences, Shanghai, China) *in vivo*. SW620 cells (5×10^6^/100 µL) were injected subcutaneously into the lower right flank of female nude mice to establish the tumor xenograft model. The tumor volume (V), which was based on caliper measurements, was calculated using the formula V (mm^3^) = length (mm)×width (mm)^ 2^/2. After the tumors reached 100 to 130 mm^3^ in size, the mice were randomly divided into control and treatment groups (n = 8). The treatment groups were administrated intratumorally at the consecutive daily doses of 5×10^8^ plaque-forming units (PFU)/100 µL of either ONYX-015, Ad·(EGFP)·CEA·E1A(Δ24), Ad (ST13)·CEA·E1A(Δ24) for four days. The control group was treated with consecutive intratumoral injections four times with the same volume of PBS.

**Figure 6:**
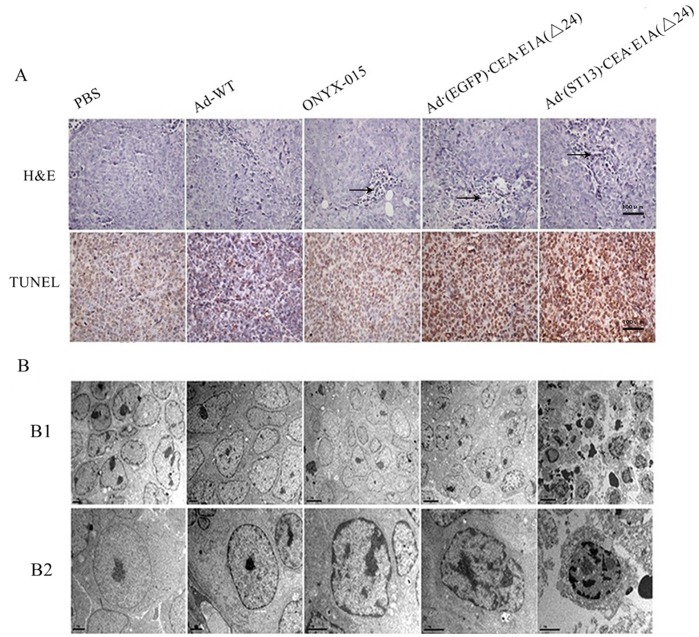
The detection of necrosis and apoptosis *in vivo.* A. Tumor sections of HCT116 CRC were analyzed for necrosis by hematoxylin–eosin (H&E) staining, for apoptosis by TdT-mediated dUTP-biotin nick-end labeling (TUNEL), and for morphological observations by transmission electron microscope (TEM) analysis. (**B1**), Apoptosis in tumor cells. (**B2**), For clarity, a single tumor was observed for apoptosis.

### Immunohistochemical and Histopathologic Experiments

For the immunohistochemical evaluation, two mice per group were randomly selected 4 days after viral administration. Under aseptic conditions, the tumor tissues were harvested and cut into pieces of approximately 1 cubic millimeter in size. The fresh tissue was immediately immersed into 4% paraformaldehyde, where it was kept for 48 h at room temperature and then embedded into paraffin. Afterward, the samples were cut into 4-µm-thick sections. Immunohistochemistry was performed with an anti-adenoviral hexon or anti-ST13 antibody (Biodesign International, Saco, ME) using an immunohistochemistry kit according to the manufacturer’s protocol. In addition, pathological changes in the tumor tissue were examined after hematoxylin and eosin (H&E) staining and TUNEL staining as well as by transmission electric microscopy (TEM).

### Statistical Analysis

All data are presented as the mean ± SD and were processed using the SPSS 10.1 statistical software. Each quantitative experiment was carried out at least three times, and statistical significance was assigned for P values ≤0.05.

**Figure 7:**
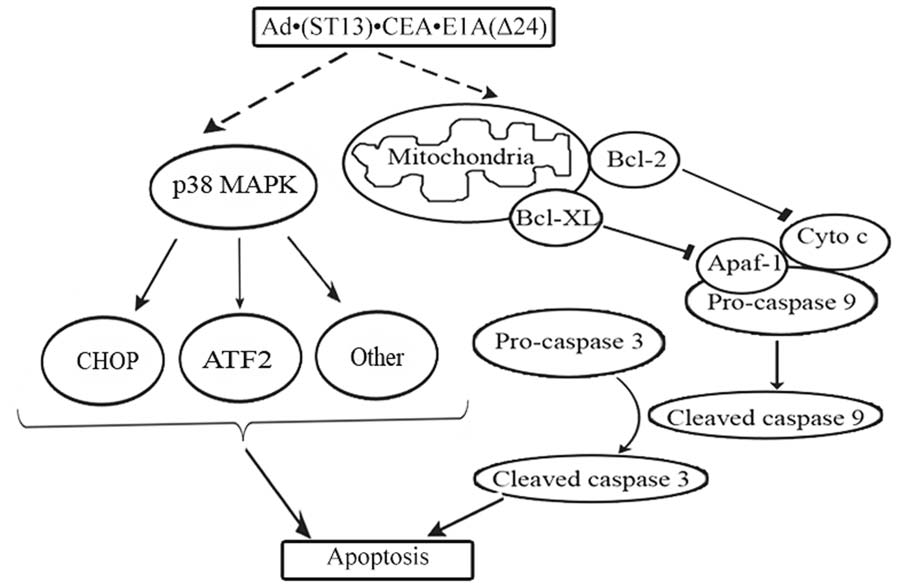
Possible mechanisms of the antitumor effect of Ad·(ST13)·CEA·E1A(Δ24) *in vivo*. After Ad·(ST13)·CEA·E1A(Δ24) infection, on the one hand, the phosphorylated P38, ATF2 and upregulation of CHOP expression were detected. On the other hand, executioner caspase-3 was activated.

## Results

### Construction and Characterization of Ad·(ST13)·CEA·E1A(Δ24)

The Ad·(ST13)·CEA·E1A(Δ24) vector was successfully constructed by replacing the native E1A promoter with the colorectal cancer-specific CEA promoter, deleting 24 bp in Ad·E1A (923–946 bp) and harboring the antitumor ST13 gene, as shown in Fig. 1A. The identification of ST13 and CEA expression by PCR was shown in Fig. 1B. To determine the E1A(Δ24) and ST13 expression of the various viruses, the CRC SW620 cell line was infected with either Ad·(ST13)·CEA·E1A(Δ24), Ad·(EGFP) CEA·E1A(Δ24), or the typical oncolytic virus ONYX-015 at an MOI of 5. Western blot analyses were used to detect E1A(Δ24) and ST13 protein. The results showed that the Ad·(ST13)·CEA·E1A(Δ24) vector induced specific ST13 expression and the greatest E1A(Δ24) expression (Fig. 1C) in detectable CRC cells.

### CRC-specific Antitumor Effect of Ad·(ST13)·CEA·E1A(Δ24) *in vitro*

CEA-positive CRC cell lines (SW620, HCT116, and HT29), and three CEA-negative cancer cell lines (breast cancer Bcap37 cell line, Nasopharynageal carcinoma CNE cell line and cervical carcinoma HeLa cell line) and two normal cell lines (QSG7701 and WI38) were infected with either Ad·(ST13)·CEA·E1A(Δ24), Ad·(EGFP)·CEA·E1A(Δ24), or ONYX-015 at the indicated MOIs (0.1, 1, 5, or 10). After 96 h, cell viability was analyzed using the MTT assay.

As shown in Fig. 2A, the oncolytic effect of Ad (ST13)·CEA·E1A(Δ24) treatment demonstrated a superior antitumor effect than did the treatment with Ad·(EGFP)·CEA·E1A(Δ24) or ONYX-015 at an MOI of 5 or 10. Furthermore, the inhibition was dose-dependent. The Bcap37, CNE and HeLa cells showed a lower level of inhibition than the three CRC cell lines, and there was no inhibition in the QSG7701 or WI38 normal cell lines.

As shown in Fig. 2B, a time course for the treatment with the recombinant viruses was also tested. Cells were infected with either Ad·(ST13)·CEA·E1A(Δ24), Ad·(EGFP)·CEA·E1A(Δ24) or ONYX-015 at an MOI of 10 for different lengths of time (24, 48, 72, or 96 h), and the cell viability after infection was determined using the MTT assay. The results indicated that cellular inhibition was time-dependent. The antitumor effect following Ad (ST13)·CEA·E1A(Δ24) treatment was superior to that following Ad·(EGFP)·CEA·E1A(Δ24) and ONYX-015 treatment in each of the cell lines examined (Fig. 2B). After 96 h, the viability of Ad·(ST13)·CEA·E1A(Δ24)-infected cells was significantly decreased. Again the cytotoxicity of the Ad·(ST13)·CEA·E1A(Δ24) on three colorectal cancers showed greater antitumor effect than that of three CEA-negative cancer, while no cytotoxicity in two normal cells.

These results indicated that Ad·(ST13)·CEA·E1A(Δ24) exerted a greater specific antitumor effect on three CEA-positive colorectal cancer cells than that of three CEA-negative cancer.

To further confirm if the antitumor effect of Ad (ST13)·CEA·E1A(Δ24) was CEA-specific or colon-specific, we compared its effect on CEA-negative colon cancer cell line (Colo-320) and CEA-positive non-colon cancer cell line (A549, MCF-7), as shown in Fig. 3C. Our findings suggested that Ad (ST13)·CEA·E1A(Δ24) was more specific on CEA-positive cancer cells.

**Morphological changes and apoptosis induced by virus treatment and assayed flow eytometry**Morphological changes in the tumor cells and normal cells treated with various viruses at an MOI of 10 after 72 hours were observed by microscopy. As shown in Fig. 3A, a cytopathic effect was observed in the CEA-positive colorectal cancer cells infected with either Ad·(ST13)·CEA·E1A(Δ24), Ad·(EGFP)·CEA·E1A(Δ24) or ONYX-015 compared with the CEA-negative HeLa cells. The results indicated that there were more significant morphological changes in Ad·(ST13)·CEA·E1A(Δ24)-infected cancer cells than in Ad·(EGFP)·CEA·E1A(Δ24)-infected or ONYX-015-infected cells, such as cell shrinkage and the appearance of small cellular fragments. Furthermore, no morphological changes were observed in the normal QSG7701 hepatic cell line. These results were further confirmed by the results of MTT assays.

To determine whether apoptosis was involved in Ad·(ST13)·CEA·E1A(Δ24)-induced cell death, apoptosis was evaluated using flow cytometry assay. SW620 cells were infected with either ONYX-015 or Ad·(ST13)·CEA·E1A(Δ24) at an MOI of 5 for 48 h. The cells were then subjected to annexin V staining to identify early-stage apoptosis and PI staining to identify late-stage apoptosis by flow cytometric analysis. As shown in Fig. 3B, the percentages of Ad·(ST13)·CEA·E1A(Δ24)-infected tumor cells in early-stage and late-stage apoptosis were 7.13% and 22.22%, respectively. The sums of the cell percentages for early- and late-stage apoptosis, which are shown in Fig. 3C, were 29.35% for Ad·(ST13)·CEA·E1A(Δ24), 8.85% for ONYX-015 and 3.9% for mock-infected cells. These data revealed that Ad (ST13)·CEA·E1A(Δ24) treatment could efficiently induce cancer cell death by specifically inducing apoptosis.

### Apoptosis Detected by Caspase Related Enzyms

Apoptosis is commonly accompanied by dramatic changes in the levels of caspase-related enzymes and proteins. Previous research had shown that ZD55-ST13 treatment induced apoptosis via the mitochondrial pathway [18]. Therefore, several apoptosis-related proteins from SW620 cells were analyzed using western blot. As shown in Fig. 4A, the level of the anti-apoptotic protein Bcl-XL was decreased, which would support the role for mitochondrial apoptosis. In addition, cleaved caspase-9, cleaved caspase-3 and the cleavage of PARP, were all markedly increased in Ad·(ST13)·CEA·E1A(Δ24)-infected cells. It was clear that Ad·(ST13)·CEA·E1A(Δ24) treatment induced apoptosis more effectively than that treatment with either Ad·(EGFP) CEA·E1A(Δ24) or ONYX-015.

The p38 signal transduction pathway, a mitogen-activated protein kinase (MAPK) pathway, plays an essential role in regulating many cellular processes, including inflammation, cell differentiation, cell growth and death. In addition, p38 also transduces signals to other cellular components for the execution of various cellular responses. ATF2, a substrate for p38, can form heterodimers with members of the Jun family of transcription factors and can thereby directly associate with the AP-1 binding site [24]. CHOP, a member of the C/EBP family of transcription factors, is also referred to as growth arrest and DNA damage-inducible gene 153 (GADD153) and is involved in the regulation of cell growth and differentiation [25]. As shown in Fig. 4B, the expression of phosphorylated p38 was significantly increased after Ad·(ST13)·CEA·E1A(Δ24) treatment. Meanwhile, activated p38 increased the level of phosphorylated ATF2 and the expression of CHOP. These results indicated that p38 may be involved in an apoptotic pathway that was induced by the CRC specific oncolytic adenovirus harboring ST13 (CTGVT-CRC). The similar results about apoptosis-related proteins were detected in human colorectal cancer cell lines HCT116. (Fig. S1).

### Antitumor Efficacy of Ad·(ST13)·CEA·E1A(Δ24) in Nude Mice

The SW620 xenograft model for human colorectal tumors was established in athymic nude mice to assess the potential antitumor efficacy of Ad·(ST13)·CEA·E1A(Δ24) *in vivo.* As shown in Fig. 5A, the tumors grew rapidly in the PBS-treated group, whereas various degrees of tumor growth suppression were observed in the ONYX-015-, Ad·(EGFP)·CEA·E1A(Δ24)- and Ad (ST13)·CEA·E1A(Δ24)-treated groups. The average volume of the Ad·(ST13)·CEA·E1A(Δ24)-treated SW620 tumors was approximately 170 mm^3^ at 30 days after treatment, which represented an increase of only 40–70 mm^3^ compared with the initial tumor volume of 100–130 mm^3^. The final tumor volume of the PBS-treated group was approximately 2500 mm^3^, indicating that there was approximately a 98% tumor growth inhibition rate in these animals, which would suggest that an almost complete inhibition was observed in the experimentally treated animals. This potent and specific inhibition of CRC using the CTGVT-CRC strategy had not previously been reported.

Additionally, animal survival was monitored until 54 days after treatment, and all the mice survived in Ad·(ST13)·CEA·E1A(Δ24) group. In the other groups, the survival rates were significantly decreased to different extents, as the Ad·(EGFP)·CEA·E1A(Δ24) group was 50% survival, the ONYX-015 group was 37.5% survival, and the PBS-treated group had only a 12.5% survival rate (Fig. 5B). These results suggested that the CTGVT-CRC strategy using Ad·(ST13)·CEA·E1A(Δ24) could efficiently improve the survival rates of mice.

To study the mechanism of Ad·(ST13)·CEA·E1A(Δ24) action, the immunohistochemical study for hexon expression of the adenovirus and the ST13 gene expression level *in vivo* were carried out. As shown in Fig. 5C, in the Ad·(ST13)·CEA·E1A(Δ24)-treated group, the hexon protein level was greater than in the wild-type virus-treated groups, and ST13 was similarly highly expressed. Thus, we assumed that the potent antitumor effect *in vivo* was due to the CEA-controlled oncolytic adenovirus and the tumor suppressive effect of the ST13 gene.

### Detection of Necrosis and Apoptosis Induced by Ad·(ST13)·CEA·E1A(Δ24) *in vivo*

To explore the viral-induced pathological changes *in vivo*, tumor samples were collected and examined using hematoxylin-eosin (H&E) staining. As shown in Fig. 6, small, necrotic foci were seen in the PBS-treated group. However, in the Ad (ST13)·CEA·E1A(Δ24)-treated group, many wide areas of necrosis were observed. The H&E staining revealed that this treatment effectively led to tumor necrosis *in vivo*. Additionally, TUNEL staining within the solid tumor demonstrated that the apoptosis mediated by the Ad (ST13)·CEA·E1A(Δ24) treatment was more prominent than that of the Ad (EGFP)·CEA·E1A(Δ24) or ONYX-015 treatments. No apparent apoptosis occurred in the Ad-WT-treated groups (Fig. 6A).

Significant levels of apoptosis were also detected by transmission electron microscopy (TEM) in the Ad·(ST13)·CEA·E1A(Δ24)-treated group and the other virus-treated groups. Furthermore, this analysis revealed that during early-stage apoptosis, there were apoptotic morphological changes, such as chromatin condensation and margination, whereas during late-stage apoptosis, there were broken nuclei, apoptotic body formations and vacuolizations of the cytoplasm that were visible (Fig. 6B).

## Discussion

Although the CTGVT (OV-gene) is a potent antitumor strategy, many modifications have still been made by us. (1) By the combination of two antitumor gene, we initiated the Cancer Targeting Dual Gene-Viro-Therapy (CTGVT-DG), and many xenografted tumors have been completely eradicated [26], [27], [28], [29], [30], [31]. By the use of this strategy that we will be sure to construct drugs with higher antitumor effect than that of 1 billion USD product OncoHSV-GM-CSF [9] and the Nature paper product OncoPox-GM-CSF [10]. (2) The tissue (organ) specific CTGVT was engineered and developed. Therefore the liver cancer specific CTGVT (CTGVT-LC) was constructed [32], [33], [34], and the prostate cancer specific CTGVT (CTGVT-PCa) was published in PLoS ONE by Dr. Ding [35]. In this report, we took several strategies to make viruses replicate selectively in CRC cells not in normal cells. The first strategy involved the deletion of a 24bp in the E1A region that was necessary for viral replication in normal cells but not in tumor cells. The other strategy was to use CRC selective promoter CEA to control the expression of E1A(Δ24). Moreover, beyond that, CRC antitumor gene ST13 was introduced to the system to improve the therapeutic efficacy of recombination viruses.

It had been reported that CEA was over-expressed in 90% of colorectal cancer cells [36]. The data suggest that CEA could be applied to improve the tumor specificity of gene expression. Sagawa *et al*. substantiated the selective replication of AdCEAp/Rep in CEA-producing tumor cell lines, in which E1A is driven by the CEA promoter. The titers of the virus were more than 1×10^7^ PFU/10^5^ cells for the CEA-producing cell lines (M7609, BxPC3, MKN45, and HT-29), whereas virus was not detectable in the non-CEA-producing cell lines (Hc and P5) [23]. Moreover, it was also shown that there were selective cytotoxic effects of AdCEAp/Rep on the CEA-producing cancer cell lines but not on the non-CEA-producing cells. To this end, we constructed a conditionally replication-competent adenovirus (CRAd) with CEA promoter to express ST13, the Ad·(ST13)·CEA·E1A(Δ24), which has E1A(Δ24) targeting to Rb-dysfunctional tumors.

It has been shown that the ST13 protein is a cytoplasmic molecule with an apparent *M*r of 50,000. The expression level of this protein is significantly downregulated in colorectal cancer, and increased of ST13 protein expression can suppress the proliferation of colorectal cancer cells. Our recent findings suggest that overexpression of ST13 gene can inhibit the growth of colorectal cancer cells [18], [19]. In this paper, the Ad·(ST13)·CEA·E1A(Δ24) vector induced specific ST13 expression, significantly inhibited the growth of xenograft SW620 colorectal carcinomas in nude mice, and prolonged the survival time in the mice. ST13 is also referred to as Hip (Hsp70 interacting protein), p48 (progesterone receptor-associated p48 protein), Hop (Hsp70-Hsp90 organizing protein) and SNC6. The ST13 may be involved in various types of cancers by regulating the functions of different target proteins through cellular chaperone/co-chaperone pathways [37], [38]. Our results showed that Ad·(ST13)·CEA·E1A(Δ24) caused the apoptosis by P38 MAPK which upregulated CHOP and ATF-2 expression, and the mitochondrial pathway based on the increase of caspase 9, caspase 3 expression(Fig. 7).

The main features of this study are:

1. Ad·(ST13)·CEA·E1A(Δ24) has been constructed which is a CRC Specific Targeting Gene-Viro-Therapy (CTGVT-CRC) with antitumor effect for three CRC specific cancers higher than that of three CEA-negative cancers while no toxicity to normal cells (Fig. 2A, B). This CTGVT-CRC has not been reported before.
2. Ad·(ST13)·CEA·E1A(Δ24) has potent antitumor effect which has 98% inhibitory rate of CRC growth without any nude mice death in the Ad·(ST13)·CEA·E1A(Δ24) treated group (Fig. 5A, B).
3. The mechanism of action for Ad·(ST13)·CEA·E1A(Δ24) is unique. The apoptosis was mediated by the P38 MAPK signaling pathway to increase the level of phosphorylated P38 and its substrate ATF2 as well as upregulation of CHOP expression. The anti-apoptotic gene Bcl-XL was down regulated and the expression of caspase 9, 3 and the cleavage of PARP were up regulated (Fig. 4A, B) which mean the apoptosis was mediated by the mitochondrial pathway.

By the way, many other modifications are going to be studied, for example, the specific targeting and killing cancer stem cells (CSC), a CTGVT-CSC will be constructed and so on.

## Supporting Information

Figure S1 **Detection of apoptosis in HCT116 cells by western blot.** HCT116 cells were infected with either ONYX-015, Ad·(EGFP)·CEA·E1A(Δ24) or Ad·(ST13)·CEA·E1A(Δ24) at an MOI of 5, for 48 h, the apoptosis-related proteins were analyzed by western blot.
(TIF)
